# TLR9-induced interferon β is associated with protection from gammaherpesvirus-induced exacerbation of lung fibrosis

**DOI:** 10.1186/1755-1536-4-18

**Published:** 2011-08-02

**Authors:** Tracy R Luckhardt, Stephanie M Coomes, Glenda Trujillo, Joshua S Stoolman, Kevin M Vannella, Urvashi Bhan, Carol A Wilke, Thomas A Moore, Galen B Toews, Cory Hogaboam, Bethany B Moore

**Affiliations:** 1 Division of Pulmonary, Allergy and Critical Care Medicine, Department of Internal Medicine, University of Alabama at Birmingham Medical School, Birmingham, AL, USA; 2 Division of Pulmonary and Critical Care Medicine, Department of Internal Medicine, University of Michigan, Ann Arbor, MI 48109, USA; 3 Graduate Program in Immunology University of Michigan, Ann Arbor, MI 48109, USA; 4 Department of Pathology, University of Michigan, Ann Arbor, MI 48109, USA; 5 Stony Brook University School of Medicine, Stony Brook, NY, 11794, USA

## Abstract

**Background:**

We have shown previously that murine gammaherpesvirus 68 (γHV68) infection exacerbates established pulmonary fibrosis. Because Toll-like receptor (TLR)-9 may be important in controlling the immune response to γHV68 infection, we examined how TLR-9 signaling effects exacerbation of fibrosis in response to viral infection, using models of bleomycin- and fluorescein isothiocyanate-induced pulmonary fibrosis in wild-type (Balb/c) and TLR-9^-/- ^mice.

**Results:**

We found that in the absence of TLR-9 signaling, there was a significant increase in collagen deposition following viral exacerbation of fibrosis. This was not associated with increased viral load in TLR-9^-/- ^mice or with major alterations in T helper (Th)1 and Th2 cytokines. We examined alveolar epithelial-cell apoptosis in both strains, but this could not explain the altered fibrotic outcomes. As expected, TLR-9^-/- ^mice had a defect in the production of interferon (IFN)-β after viral infection. Balb/c fibroblasts infected with γHV68 *in vitro *produced more IFN-β than did infected TLR-9^-/- ^fibroblasts. Accordingly, *in vitro *infection of Balb/c fibroblasts resulted in reduced proliferation rates whereas infection of TLR-9^-/- ^fibroblasts did not. Finally, therapeutic administration of CpG oligodeoxynucleotides ameliorated bleomycin-induced fibrosis in wild-type mice.

**Conclusions:**

These results show a protective role for TLR-9 signaling in murine models of lung fibrosis, and highlight differences in the biology of TLR-9 between mice and humans.

## Background

Idiopathic pulmonary fibrosis (IPF) is a progressive, fibrotic lung disease with a poor survival rate. Causes of IPF may relate to epithelial-cell injury, abnormal fibroproliferation, inflammation, and deposition of extracellular matrix components [1,2]. Standard therapies have shown little benefit, and most patients progress to respiratory failure.

Many patients with IPF have a slow progressive disease course over months to years after diagnosis [3]; however, some patients experience acute deterioration in pulmonary function [3-7] without clear cause. This is referred to as acute exacerbation of IPF. Histological findings are diffuse alveolar damage or organizing pneumonia plus usual interstitial pneumonitis [4-7]. Reported mortality rates are often greater than 78% in this population of patients [7].

Viral infections have been linked to the development of fibrosis in both human and animal studies [8-13]. We have shown previously that γHV68 infection augments fluorescein isothiocyanate (FITC)-induced pulmonary fibrosis when given 14 days after fibrotic challenge [14]. This exacerbation of fibrosis caused by γHV68 shares many similarities with acute exacerbations in humans, including decreased lung compliance and diffuse alveolar damage on histopathology [14].

The pathogenesis of IPF is unclear. Many cell types have been implicated in the pathogenesis of the fibrotic response, including mesenchymal cells [15-17] and alveolar epithelial cells (AECs) [18]. AECs are closely approximated to the mesenchymal (fibroblast) cells within the lung, and are believed to play an important role in homeostasis between epithelial and mesenchymal cells. AEC injury is a universal feature seen in the pathobiology of fibrotic lung disease [19], and there is good evidence that AEC apoptosis plays a role in the pathogenesis of pulmonary fibrosis [20-22]). A role for gammaherpesviral infection in the induction of AEC apoptosis has been suggested. Egan *et al*. reported that, compared with controls, patients with IPF had increased Epstein-Barr virus (EBV) DNA loads in lung tissue, and some of these patients also had positive staining for p53, suggesting an increase in apoptosis [10]. Additionally, *in vitro *infection of human lung epithelial cells with EBV induced secretion of transforming growth factor (TGF)-β1 secretion and upregulated caspases 3 and 7 [23]. However, mechanistic studies using animal models have not been carried out.

The human immune system has a series of 10 innate immune receptors known as Toll-like receptors (TLRs), which enable host cells to recognize foreign pathogens. TLR-9 is an endosomal receptor that recognizes unmethylated CpG nucleotides, which are commonly found in bacterial and viral DNA genomes [24]. Stimulation of TLR-9 results in activation of the MyD88 pathway, resulting ultimately in the activation of Jun N-terminal kinase and the translocation of nuclear factor (NF)κB to the nucleus [24]. TLR-9 stimulation is associated with the development of T helper (Th)1 immune responses [25,26]. TLR-9 is expressed at its highest levels in plasmacytoid dendritic cells (DCs) and B cells [24], but it has also been found on lung epithelial cells [27] and fibroblasts [28]. TLR-9 has been shown to be important in the pathogenesis of γHV68, with TLR-9^-/- ^mice being more susceptible to both lytic and latent γHV68 infection after intraperitoneal inoculation [29].

In this paper, we show that TLR-9 signaling can limit the exacerbation of the fibrotic response by γHV68 infection in the lung. This exacerbation is not associated with increased epithelial-cell apoptosis or major alterations in Th1 or Th2 cytokines, but rather, viral infection in wild-type, but not TLR-9^-/- ^mice, leads to production of IFN-β and diminished fibroproliferation. Furthermore, therapeutic administration of CpG oligodeoxynucleotides (ODN) can limit bleomycin-induced pulmonary fibrosis.

## Results

### Toll-like receptor-9 signaling protects mice from viral-induced exacerbation of fibrosis, but has no effect on fibrotic insults alone

**Figure 1:**
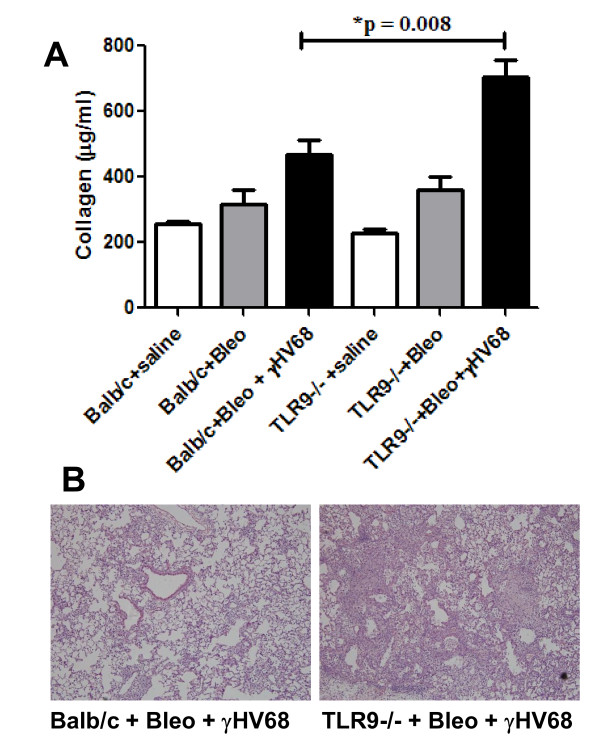
**TLR-9^-/- ^mice are more susceptible to viral exacerbation of fibrosis**. **(A) **Balb/c and TLR-9^-/- ^mice were treated with either saline or bleomycin intratracheally on day 0. They were then given either sham infection or infection with 5 × 10^4 ^PFU of γHV68 on day 14 by intranasal infection. On day 21, lungs were harvested and Sircol assay was performed. TLR-9^-/- ^mice were more susceptible than wild-type mice to viral exacerbation of existing fibrosis (705.4 *± *52.79 versus 468.6 *± *42 mg collagen/ml; n = 5; *P *< 0.01). There were no significant differences in collagen levels between Balb/c and TLR-9^-/- ^saline-treated mice (255 *± *9.06 versus 227.3 *± *14.95 mg/ml) or between both groups after bleomycin challenge alone (317.0 *± *43.0 versus 362.5 *± *78.8 mg/ml.) This was true in three experiments, and the relative increase in collagen production in bleomycin-treated mice over saline controls was also not different. **(B) **Lungs from WT or TLR-9^-/- ^mice treated with bleomcyin on day 0 and infected with γHV68 on day 14. Lungs were harvested for histologic analysis on day 21 and stained with hematoxylin and eosin. Sections are representative of three mice examined.

Balb/c and TLR-9^-/- ^mice were treated with either saline or bleomycin on day 0. On day 14, a time of established pulmonary fibrosis [30], the mice were given either γHV68 infection (5 × 10^4 ^plaque-forming units (PFU)) or Sham infection (PBS) intranasally. On day 21, a time point that represents 7 days after infection in the fibrotic mice, the lungs were harvested. Collagen content was determined by Sircol assay (Figure 1A). Baseline collagen levels between Balb/c and TLR-9^-/- ^mice were similar, and the mice had similar increases in collagen levels with bleomycin treatment alone. However, the TLR-9^-/- ^mice had a greater increase in collagen on day 21 after viral infection than did wild-type mice (705.4 *± *52.8 versus 468.6 *± *42.2 μg/ml; *P *< 0.01; n = 5). On histopathological examination, areas of alveolar fibrosis and consolidation were found to be increased in TLR-9^-/- ^compared with Balb/c mice after fibrotic challenge and viral infection (Figure 1B). Similar trends were seen with mice treated with FITC followed by γHV68 or Sham infection (see Additional file 1, supplemental Figure 1), although the difference between Balb/c and TLR-9^-/- ^mice treated with FITC plus γHV68 did not reach significance.

### Fibrotic mice are more susceptible to viral replication, but Toll-like receptor-9 deficiency does not alter replication in the lungs

**Figure 2:**
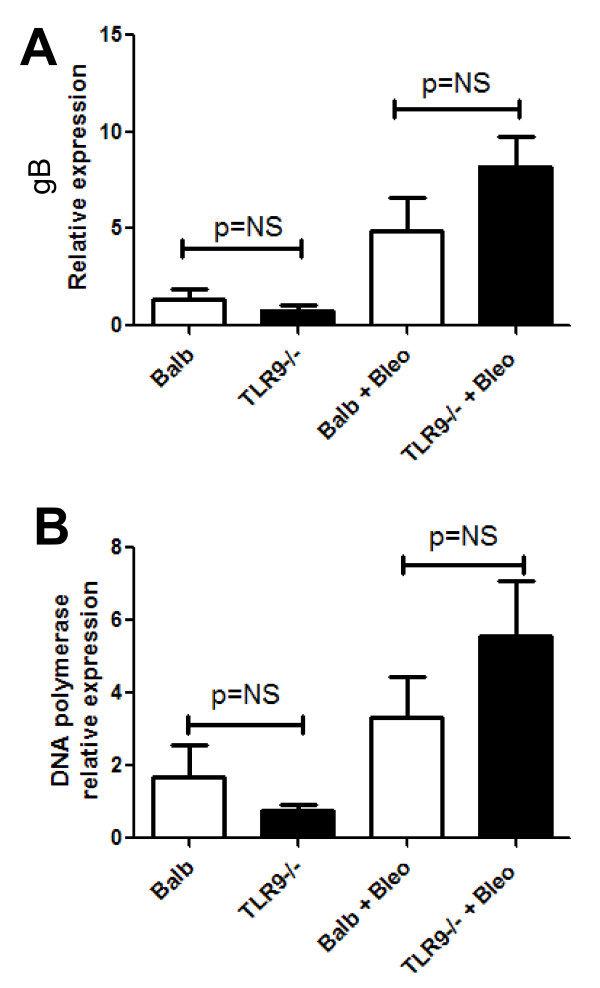
**Viral replication is not different in the lungs of Balb/c and TLR-9^-/- ^mice**. Balb/c or TLR-9^-/- ^mice were injected with saline or bleomycin intratracheally on day 0. On day 14, mice received 5 × 10^4 ^PFU gamma herpesvirus 68 (γHV68) intranasally. On day 21, lungs were harvested, and total RNA was prepared and analyzed for expression of **(A) **the viral envelope glycoprotein gene (gB) or **(B) **viral DNA polymerase. A single mouse in the Balb/c group not treated with bleomycin was normalized to 1, and all other mice were plotted relative to this control (n = 5 mice/group, representative of two experiments). The ΔC_T _for each group are as follows: For the gB gene: Balb 4.9 *± *0.5, TLR-9^-/- ^5.7 *± *0.5, Balb plus bleomycin 3.07 *± *0.6, TLR-9^-/- ^plus bleomycin 2.8 *± *0.7. For the DNA polymerase gene: Balb 4.5 *± *0.6, TLR-9^-/- ^5.4 *± *0.4, Balb plus bleomycin 3.3 *± *0.6, TLR-9^-/- ^plus bleomycin 2.8 *± *0.8.

Guggemoos *et al*. reported previously that TLR-9^-/- ^mice were more susceptible to intraperitoneal γHV68 infection, but were not more susceptible to intranasal infection [29]. To determine whether fibrotic challenge increased viral replication, Balb/c or TLR-9^-/- ^mice were infected with 5 × 10^4 ^PFU γHV68 alone or on day 14 after bleomycin challenge. Lungs were harvested 7 days after viral infection, and expression levels of the envelope glycoprotein B mRNA (Figure 2A) or the viral DNA polymerase mRNA (Figure 2B) were measured by real-time reverse transcription (RT)-PCR. Expression of both viral transcripts was increased in bleomycin-exposed lungs compared with naive lungs. However, TLR-9 deficiency did not worsen the viral replication in the lung compared with Balb/c mice, consistent with previous findings [29].

In our experience, measurement of lytic viral load by RT-PCR correlates well with viral titers determined by plaque assay [14,31].

### Alveolar epithelial cells, fibroblasts and fibrocytes all express Toll-like receptor-9

**Figure 3:**
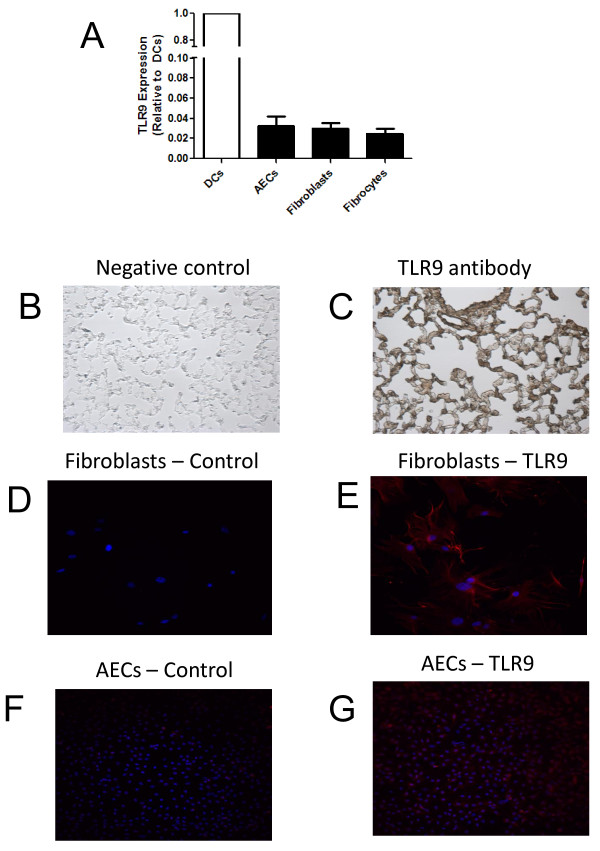
**Alveolar epithelial cells (AECs), fibroblasts and fibrocytes all express Toll-like receptor-9**. AECs, fibroblasts, fibrocytes and whole lungs were isolated from Balb/c, mice and total RNA prepared and analyzed for TLR-9 expression. The data were normalized to the expression level of TLR-9 in dendritic cells, which was set to 1 (n = 3/group). No TLR-9 message was amplified in cells from TLR-9^-/- ^mice. **(B, C) **Immunohistochemistry was performed on paraffin wax-embedded whole-lung tissue from normal Balb/c mice. **(B) **Control staining with secondary antibody only; **(C) **diffuse TLR-9 staining, with notable staining in the bronchial epithelium, at the alveolar junctions and the interstitium. Fibroblasts **(E) **and AECs **(G) **were isolated from Balb/c mice and fixed. Immunofluorescence staining for TLR-9 shows that both cell types expressed TLR-9. The control slides with secondary antibody alone **(D and F) **did not show any auto-fluorescence or non-specific staining.

We have shown previously that alveolar epithelial cells and mesenchymal cells are target cells for viral infection within the lung [31,32]. Furthermore, lung epithelial cells [27] and fibroblasts [28) have been reported to express TLR-9. Because these cell types are known to be important in the pathogenesis of fibrosis, we sought to verify whether TLR-9 was expressed in these cells. The expression level of TLR-9 was measured by real-time RT-PCR in isolated AECs, fibroblasts and fibrocytes and compared with expression levels in DCs, which were normalized to 1 (Figure 3). Our results confirm that all three of these cell types express TLR-9, albeit with mRNA levels that are about 30-fold lower than in DCs. There was diffuse expression of TLR-9 in lung sections from Balb/c mice (Figure 3C), with TLR-9 staining in the bronchial epithelium; at the alveolar junctions, where there are likely to be type II AECs; and in the interstitium, where fibroblasts are located. TLR-9 expression was also evaluated in fibroblasts and type II AECs isolated from Balb/c mice (Figure 3E,G).

### Apoptosis of alveolar epithelial cells does not differ between Balb/c and TLR9^-/- ^mice treated with bleomycin and gammaherpesvirus 68

**Figure 4:**
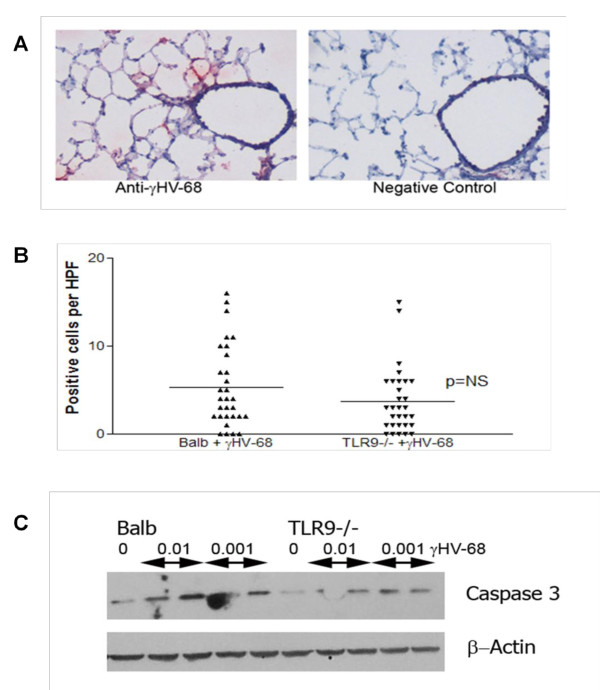
**Gamma herpesvirus 68 (γHV68) replicates in alveolar epithelial cells (AECs)**. **(A) **Wild-type mice were infected with 5 × 10^4 ^PFU γHV68 on day 0. On day 7 after infection, frozen sections were prepared, and stained with a rabbit polyclonal antisera against γHV68, or with non-immune rabbit sera as control. The goat anti-rabbit secondary was linked to alkaline phosphatase. Vivid replication of γHV68 is visible in alveolar lining cells (original magnification × 100). Sections shown are representative of four mice examined. **(B) **AECs were isolated from lungs of Balb/c or TLR-9^-/- ^mice treated with bleomycin plus γHV68 on day 21, and were cultured on fibronectin-coated slides (TiterTek). Sections were stained with antibodies (M30 Cytodeath), and the number of positive cells per high power field (HPF; ×400) were calculated (n = 30 HPF per genotype). **(C) **AECs were isolated from Balb/c or TLR-9^-/- ^mice and cells were infected *in vitro *with 0.01 or 0.001 PFU γHV68 for 48 hours. Cell lysates were then analyzed for cleaved caspase 3 by western blotting. Data are from one experiment, representative of two.

Intranasal infection with γHV68 resulted in viral infection and replication that was evident in alveolar lining cells, which probably include both type 1 and type 2 epithelial cells (Figure 4A). To determine whether the viral-induced exacerbation in TLR-9^-/- ^mice was related to alterations in AEC apoptosis, we purified AECs from mice that had been treated with both bleomycin and γHV68. Assessment of the purity of these AEC preps showed greater than 95% for e-cadherin (an epithelial-cell marker) and they contained less than 3% contamination by vimentin-positive cells (fibroblasts and macrophages) (see Additional file 1, supplemental Figure 2). These isolated cells were cultured in Lab-Tek chamber slides (#177402, Nunc, Rochester, NY) and stained with an epithelial cell-specific apoptosis antibody that recognizes a caspase cleavage product of cytokeratin 18 (M30), then the number of positive cells per high power field (HPF) was determined. There was an equivalent level of apoptosis in epithelial cells isolated from each strain (Figure 4B).

AECs were also isolated from naïve Balb/c or TLR-9^-/- ^mice, and these cells were infected with either 0.01 or 0.001 MOI (multiplicity of infection) of γHV68 for 48 hours, before cell lysates were prepared and analyzed for cleaved caspase 3 by western blotting. Viral infection induced caspase 3 activation in both genotypes, but there was no evidence of increased caspase 3 activation in the TLR-9^-/- ^cells compared with the Balb/c cells (Figure 4C). Thus, the reason TLR-9^-/- ^mice develop worse fibrosis does not appear to be related to alterations in AEC apoptosis.

### Balb/c and TLR9^-/- ^alveolar epithelial cells are equivalent in their ability to suppress fibroproliferation

To determine whether there was an inherent difference in the ability of AECs from Balb/c and TLR-9^-/- ^mice to limit fibroproliferation, AECs were isolated from either Balb/c or TLR-9^-/- ^mice, and these cells were co-cultured with fibroblasts from either Balb/c mice (see Additional file 1, supplementary Figure 3A) or with fibroblasts from TLR-9^-/- ^mice (see Additional file 1, supplementary Figure 3B). AECs from both strains were equivalent in their ability to limit fibroproliferation over a 24 h period.

### TLR9^-/- ^mice treated with bleomycin plus gammaherpesvirus 68 show increased CD8 recruitment

**Figure 5:**
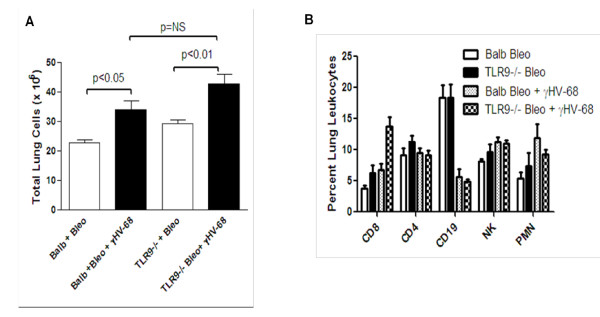
**Inflammatory responses are similar in Balb/c and TLR-9^-/- ^mice**. Mice were treated with bleomycin on day 0, and were given 5 × 10^4 ^PFU γHV68 intranasally on day 14. On day 21, lungs were collected and subjected to digestion with collagenase and DNAse. **(A) **Lung leukocytes were enumerated (n = 4 mice/group, representative of four experiments). **(B) **Isolated lung leukocytes were examined by flow cytometry for the percentage surface expression of CD4, CD8, CD19, Gr-1 (PMNs) and DX5 (NK cells). Samples were gated on CD45+ cells first. Percentages of lymphocytes were then determined within a lymphocyte gate. Gr-1 percentages were gated on CD45+ non-lymphocyte sized cells (n = 4/group representative of two experiments). **P < 0.01 for CD8 in TLR-9^-/- ^mice treated with bleomycin plus γHV68 compared with Balb/c with same treatment. For B cells, virally infected mice were significantly different from mice treated with bleomycin alone in both genotypes (*P *< 0.01).

To determine the magnitude and composition of the inflammatory response that occurs after bleomycin with or without γHV68 infection, mice of both genotypes were treated with bleomycin on day 0. On day 14, mice were either infected with γHV68 or were sham-infected. On day 21, lungs were collected, and digested with collagenase and DNAse to isolate lung leukocytes. Viral infection stimulates an inflammatory response that was greater than that noted in mice treated with bleomycin alone (Figure 5A). However, there was no difference in the magnitude of the inflammatory-cell influx between Balb/c and TLR-9^-/- ^strains. When particular cell types were analyzed by flow cytometry, it was found that after infection, TLR-9^-/- ^mice had a higher percentage of CD8+ T cells in the lung, and both strains of mice had a reduction in B cells (Figure 5B). Both strains tended to have higher numbers of neutrophils after infection, as expected.

In our previous work, we found that viral exacerbation of fibrosis in wild-type mice correlated with viral-induced fibrocyte accumulation in the lung [14]. To determine whether fibrocyte recruitment was different between Balb/c and TLR-9^-/- ^mice treated with bleomycin plus γHV68, lung leukocytes were isolated by collagenase and DNAse digestion on day 21, and these cells were stained for CD45+ collagen 1+ cells. Lungs of Balb/c mice contained 1.2 *± *0.14% fibrocytes, which was not significantly different from the 2.02 *± *0.5% seen in TLR-9^-/- ^mice (n = 4, *P *= NS). Thus, differential recruitment of fibrocytes was not seen in TLR-9^-/- ^versus control mice.

### TLR9^-/- ^mice have reduced T helper 2 responses during viral exacerbation of fibrosis

Balb/c mice and TLR-9^-/- ^mice were treated with bleomycin on day 0. On day 14 the mice were given γHV68 infection (5 × 10^4 ^PFU). On days 0, 14, 17 and 21, lungs were harvested, and whole-lung homogenates were assayed for Th1 and Th2 cytokines by ELISA. Because γHV68 has been shown to be fibrotic in Th2-biased mice [11], we wanted to determine whether TLR-9^-/- ^mice had increased Th2 profiles. There were modest fluctuations in the Th2 cytokines over the 21 day course, but no evidence of a Th2 skewing in the TLR-9^-/- ^mice (see Additional file 1, supplementary Figure 4A, B). In fact, when treated with bleomycin plus γHV68, the TLR-9^-/- ^mice had decreased levels of interleuking (IL)-4 and IL-13 on day 21 compared with baseline measurements; these trends were not noted in the Balb/c mice. When Th1 cytokines were analyzed, both Balb/c and TLR-9^-/- ^mice mounted strong interferon-γ responses by day 21 of the experiment, which corresponded to day 7 after viral infection (see Additional file 1, supplementary Figure 4(C). By contrast, IL-12 levels diminished after infection in both genotypes (see Additional file 1, supplementary Figure 4(D). There were few changes in expression profiles of IL-17 or tumor necrosis factor-α (see Additional file 1 supplementary Figure 4E and 4F). Additionally, when total TGF-β levels were measured after acid activation by ELISA on day 21 following bleomycin plus γHV68 infection, there was no significant difference between the genotypes (see Additional file 1, supplementary Figure 5).

### TLR9^-/- ^mice are defective in IFN-β expression post challenge

**Figure 6:**
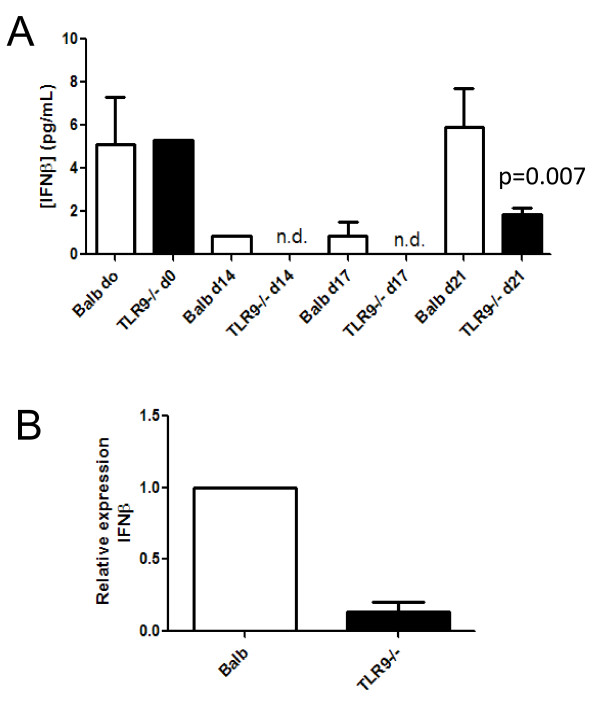
**TLR-9^-/- ^mice are defective in interferon (IFN)-β production**. **(A) **Balb/c or TLR-9^-/- ^mice were injected with bleomycin on day 0. On day 14 mice received γHV68 intranasally. Lungs were harvested on days 0, 14, 17 and 21, and lung homogenates were prepared and analyzed by ELISA for IFN-β (n = 5 mice/group). **(B) **Fibroblasts isolated from Balb/c or TLR-9^-/- ^mice were infected *in vitro *with 0.01 PFU γHV68 and total RNA was prepared after 24 h of infection for analysis of IFN-β by real-time RT-PCR. The mean of the Balb/c samples was set to 1, n = 5/group.

TLR-9 stimulation is known to be crucial for type I IFN production [33]. Thus, we sought to determine whether the TLR-9^-/- ^mice had differences in production of IFN-β after bleomycin plus γHV68 challenge. Mice were given bleomycin on day 0 and were infected with γHV68 on day 14. Lungs were harvested on days 0, 14, 17 and 21, and IFN-β expression was determined in whole-lung homogenates by ELISA. Bleomycin challenge resulted in a loss of IFN-β expression in both genotypes (Figure 6A), suggesting that this reduction is TLR-9-independent. Following viral infection on day 14, production of IFN-β was restored by day 21 (a week after viral challenge) in Balb/c mice, but there was a significant reduction in IFN-β production in TLR-9^-/- ^mice. Furthermore, when fibroblasts isolated from Balb/c or TLR-9^-/- ^mice were infected with 0.01 PFU γHV68 *in vitro *for 24 hours, and mRNA was analyzed for expression of IFN-β, expression in TLR-9^-/- ^fibroblasts was reduced nearly 10-fold (Figure 6B).

### Gammaherpesvirus 68 infection induces IFN-β production, which limits fibroblast proliferation

**Figure 7:**
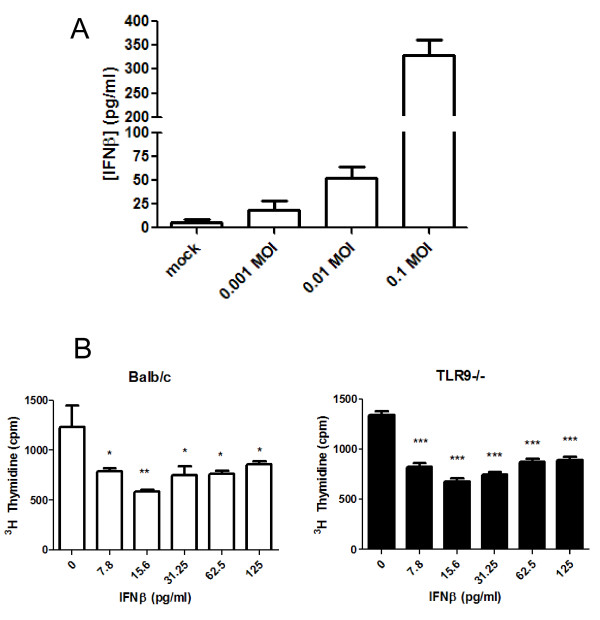
**Gamma herpesvirus 68 (γHV68) infection induces interferon (IFN)-β, which in turn limits fibroblast proliferation**. **(A) **Fibroblasts from Balb/c mice were infected with 0, 0.001, 0.01 and 0.1 MOI γHV68 *in vitro*, and levels of IFN-β in the supernatant were measured 24 hours later (n = 6/group). **(B) **Fibroblasts from (left) Balb/c or (right) TLR-9^-/- ^mice were cultured at 5000 cells/well in the presence of increasing concentrations of IFN-β. IFN-β limited proliferation of fibroblasts at all doses tested (n = 6/group).

To determine whether infected fibroblasts could produce IFN-β, Balb/c fibroblasts were cultured at 50,000 cells/well an infected with 0, 0.1, 0.01 and 0.001 MOI γHV68 for 24 h. Culture supernatants were harvested and analyzed for IFN-β production by ELISA (Figure 7A). These data show that even at low MOI, γHV68 could stimulate IFN-β production. To determine whether these concentrations of IFN-β were biologically significant, Balb/c and TLR-9^-/- ^fibroblasts were cultured at 5000 cells/well in the presence of increasing concentrations of recombinant IFN-β. Doses as low as 7.5 pg/ml were effective in limiting fibroblast proliferation in both strains (Figure 7B).

### Gammaherpesvirus 68 infection reduces proliferation of Balb/c, but not TLR9^-/- ^fibroblasts

**Figure 8:**
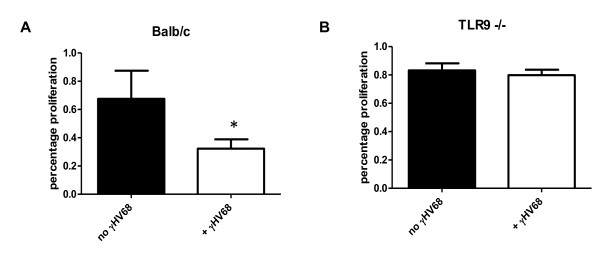
**Infection by gamma herpesvirus 68 (γHV68) limits Balb/c, but not TLR-9^-/- ^fibroblast proliferation**. Fibroblasts from **(A) **Balb/c and **(B) **TLR-9^-/- ^mice were infected *in vitro *with 0.01 PFU γHV68, and proliferation was measured 24 hours later by ^3^H-thymidine incorporation (n = 6 samples/group). The results are presented as the percentage proliferation compared with the highest value in the uninfected fibroblasts. In the wild-type mice, infection resulted in a 35.2% reduction in proliferation (67.5% *± *8.9% versus 32.3% *± *2.7%, *P *= 0.003), whereas in the TLR-9^-/- ^mice, infection did not result in a significant change in proliferation (83.2% *± *5.0% versus 79.8% *± *3.9%, *P *= 0.56).

Based on the IFN-β results (Figure 7) and the previous observations that type I interferons inhibit lung fibroblast proliferation [34], we hypothesized that viral infection of Balb/c fibroblasts would result in reduced fibroblast proliferation soon after infection. Indeed, infection of Balb/c fibroblasts with 0.01 PFU/cell γHV68 resulted in diminished proliferation when measured at 24 hours after infection (Figure 8A). By contrast, viral infection of TLR-9^-/- ^fibroblasts had no effect on proliferation within this time frame (Figure 8B). These results suggest that induction of IFN-β by viral infection in the Balb/c mice may serve to limit the degree of exacerbation caused by viral infection.

### CpG therapy can limit bleomycin-induced fibrosis

**Figure 9:**
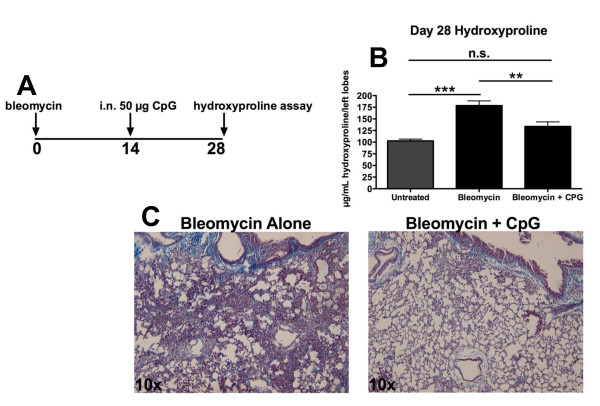
**CpG oligodeoxynucleotides (ODN) stimulation lessens bleomycin-induced fibrosis**. **(A) **C57Bl/6 male mice were injected with 0.25U bleomycin intrathecally on day 0. **(B) **On day 14, 50 μg CpG ODN was administered intranasally, and lung collagen was measured by hyroxyproline on day 28. **(C) **Lung sections were stained with Masson's trichrome to detect blue coloration indicative of collagen (n = 5 mice/group).

Our results suggested that TLR-9 stimulation during viral exacerbation of fibrosis might be protective. Thus, we investigated whether forced (non-infectious) stimulation of TLR-9 in bleomycin-induced fibrosis might also be protective. To test this, we first administered CpG ODN to the lungs of Balb/c and TLR-9^-/- ^mice, starting on day 14 after challenge, and harvested the lungs for collagen deposition on day 21. Unfortunately, in these experiments, the fibrotic response to bleomycin (from Sigma) was weaker than expected in the Balb/c background, making it difficult to ascertain whether CpG ODN treatment was effective. Thus, we repeated these experiments using the C57Bl/6 strain, which is has a more robust fibrotic response to bleomycin and also used a clinical source of the drug(Blenoxane; Bristol-Myers Squibb, New York, NY, USA) [35]. Administration of a single dose of 50 μg CpG ODN intranasally on day 14 after bleomycin (Figure 9A) attenuated the fibrotic response measured on day 28 by hydroxyproline assay (Figure 9B). Representative histology sections stained with Masson's trichrome also showed substantial improvement in the fibrotic outcome (Figure 9C).

## Discussion

We have previously shown that a gammaherpesvirus infection can exacerbate established FITC-induced fibrosis in a murine model [14]. In this study, we found that TLR-9 signaling plays a role in limiting the profibrotic exacerbations of gammaherpesviral infection. There are many potential mechanisms by which TLR-9 signaling might influence the fibrotic environment during viral infection. One possible mechanism is simply by limiting viral replication. Guggemoos *et al*. showed that TLR-9 signaling is important in control of γHV68 infection when given intraperitoneally [29]; however, they were not able to show that TLR-9 signaling in the lung was important in controlling an intranasal infection with γHV68. Our data are consistent with this finding. Although replication of the virus was greater in mice treated with bleomycin compared with non-fibrotic mice, the absence of TLR-9 had little effect on viral gene expression in either group. Although it is not entirely clear why viral replication is enhanced in fibrotic mice, it is likely that proinflammatory and profibrotic factors enhance viral gene transcription. In support of this hypothesis, prostaglandin E_2 _has been shown to promote viral replication [36]. Thus, our results confirm that TLR-9 signaling is not required for control of lytic γHV68 infection in the lung. The reasons why TLR-9 would differentially regulate lung versus peritoneal infection probably reflect the differences in the cell populations that are initially infected in each site.

Within the lung, AECs are one of the primary cell types infected with γHV68, as confirmed by our immunohistochemical findings in this study (Figure 4A) and our previous studies [31,32]. Although it is known that γHV68 expresses proteins that can prevent apoptosis and enable establishment of latent infection [37-39], previous studies in human cells and tissues have suggested a role for gammaherpesviruses in the induction of AEC apoptosis [10,23]. Furthermore, TLR-9 stimulation has been shown to inhibit macrophage apoptosis [40]. Thus, we were surprised to discover that levels of apoptosis in AECs isolated from mice treated with bleomycin plusγHV68 were similar between Balb/c and TLR-9^-/- ^genotypes. This was true regardless of whether apoptosis was assessed by M30 staining or caspase activation.

One important aspect of AEC function is to limit fibroproliferation. Profibrotic stimuli (for example, chemokine (C-C motif) ligand 2 (CCL2)) are known to alter the ability of isolated AECs to limit fibroproliferation [41]. Thus, we sought to determine whether there were basal differences in the ability of the AECs from Balb/c or TLR-9^-/- ^mice to control fibroblast proliferation. However, AECs from both genotypes of mice were equivalent in their ability to limit fibroblast proliferation from both strains. Taken together, we could not find substantial evidence of altered AEC apoptosis or function to explain the exaggerated viral-induced fibrotic response noted in TLR-9^-/- ^mice.

We next investigated the inflammatory response between Balb/c and TLR-9^-/- ^mice treated with bleomycin plus γHV68. After viral exacerbation, both genotypes of mice had an increase in total numbers of inflammatory cells, but there were few differences noted in the particular cell types recruited. There were no significant differences in fibrocyte accumulation, or in the percentages of neutrophils or CD4, natural killer or B cells between genotypes. The TLR-9^-/- ^mice did have a higher percentage of CD8+ cells, and it is possible that if activated, these CD8+ cells could contribute to tissue damage, which might exaggerate the fibrotic response in the TLR-9^-/- ^mice. Both genotypes had a loss of B cells after infection, and we believe this represents the migration of B cells to the spleen, the known major reservoir for latent viral infection [42].

Because TLR-9 signaling leads to NFκB activation and the production of Th1 cytokines [43], it seemed likely that TLR-9^-/- ^mice would have a more Th2-biased cytokine environment. Because Th2-biased mice are known to be prone to the development of fibrosis in response to infection with γHV68 [11], it was possible that a cytokine imbalance could explain the exacerbation of fibrosis in TLR-9^-/- ^mice. However, there was no evidence of Th2 skewing or defective Th1 signaling in the TLR-9^-/- ^mice during the acute response to bleomycin and infection. In fact, IL-4 and IL-13 levels were reduced on day 21 after bleomycin plus γHV68 administration in TLR-9^-/- ^mice. We were surprised that IFN-γ was not diminished in the TLR-9^-/- ^mice, and we believe that this may reflect the fact that in AECs at least, activation of other TLRs may help to stimulate NFκB activation for IFN-γ production. We confirmed that lung AECs from TLR-9^-/- ^mice express normal levels of TLR7 and TLR8, for instance (data not shown). Additionally, differences in production of TGF-β were not noted between genotypes. The observation that fibrosis is exaggerated despite strong induction of IFN-γ confirms our previous findings of viral exacerbation in the C57Bl/6 background [14]. Thus, we conclude that neither reduced production of Th1 cytokines nor increased production of Th2 cytokines in response to infection can explain the enhanced exacerbation of fibrosis noted in TLR-9^-/- ^mice.

Not surprisingly, the one cytokine whose production was different between Balb/c and TLR-9^-/- ^mice was IFN-β. TLR-9 stimulation is known to induce type I interferons [33]. Levels of IFN-β were reduced after bleomycin plus virus in whole-lung homogenates and in infected fibroblasts. It is interesting that after bleomycin administration alone, levels of IFN-β were reduced (Figure 6A). The fact that this happened in both Balb/c and TLR-9^-/- ^mice suggest a TLR-9-indepdent mechanism for the reduction in IFN-β after a fibrotic insult. This should not necessarily be interpreted to mean that bleomycin causes a reduction in TLR-9 expression, as we have no evidence of that, at least in fibroblasts isolated from fibrotic mice (data not shown). As IFN-β is known to be a potent inhibitor of lung fibroblast proliferation [34], a fact we have confirmed (Figure 7), it is reasonable to conclude that early viral infection of fibroblasts would result in the stimulation of TLR-9 by viral CpG DNA sequences and a concomitant decrease in fibroblast proliferation. In fact, we found that fibroblasts from Balb/c mice potently upregulated IFN-β secretion in response to even low-level infection. Furthermore, exogenous IFN-β could limit proliferation of fibroblast from both strains, suggesting that the defect in TLR-9^-/- ^mice is not sensitivity to, but rather production of, IFN-β. Taken together, we conclude that although viral infection after fibrotic challenge increases the fibrotic response of both strains, the more extensive exacerbation of fibrosis noted in the TLR-9^-/- ^mice is most likely due to deficiencies in IFN-β production, which in turn allow for increased fibroproliferation. When cell number and viral dose were equivalent, fibroproliferation was inhibited in Balb/c, but not TLR-9^-/- ^mice (Figure 8). Because there may be some TLR-9-independent signaling that leads to IFN-β production in the lung by day 7 after infection (Figure 6A), it is possible that at later time points, sufficient IFN-β may be available to limit fibroproliferation even in TLR-9^-/- ^mice. It is important to remember that secretion of IFN-β in the lung would probably inhibit the proliferation of other resident fibroblasts, not just those that happened to be infected.

TLR-9^-/- ^mice did not respond differently from wild-type mice to bleomycin alone or FITC alone. These results suggest that fibrotic insults alone may not generate endogenous ligands for TLR-9 stimulation. Thus, we next wanted to determine whether therapeutic stimulation of TLR-9 with CpG ODN could protect against fibrotic challenge. We initially tried to perform these experiments in the Balb/c background, but had poor responses to bleomycin in two separate experiments. Although levels of collagen were somewhat lower in CpG ODN-treated mice than in control mice, the overall levels of fibrosis were so low that meaningful interpretations of the data were not possible. Given the expense of these experiments, we chose to perform them in the C57Bl/6 background, which has a more reproducible fibrotic response to bleomycin or Blenoxane [35]. We found that mice treated intranasally with CpG ODN were significantly protected from the development of bleomycin-induced fibrosis (Figure 9). Although these results in murine studies were very exciting, recent studies in human cells have made it clear that it is unlikely that these results can be extrapolated to humans.

Our results are not the first to describe an enhanced fibrotic response in TLR-9^-/- ^mice. Earlier studies have shown that granulomas that form in response to *Schistosoma mansoni *eggs in TLR9-/- mice are larger and have more collagen deposition within the granuloma than in wild-type mice [44]. However, in that study, the results were associated with diminished Th1 and augmented Th2 responses, probably reflecting the strong Th2-skewing nature of the *S. mansoni *egg antigens, whereas we used a Th1-inducing viral infection. Thus, there are now at least two examples of TLR-9 signaling showing a protective effect in lung-fibrosis models in mice. By contrast, Meneghin *et al*. found that TLR-9 expression in human lung fibroblasts promotes myofibroblast differentiation [28], and was anticipated to worsen fibrotic disease. This human study also found that TLR-9 was expressed at low levels in surgical lung biopsies of normal subjects, but was dramatically upregulated in the biopsies from patients with fibrotic lung diseases. These data suggest that TLR-9 expression might be low in normal human lung fibroblasts, whereas our studies suggest that murine lung fibroblasts express this receptor constitutively. In addition, TLR-9 expression in human lung epithelium is modest compared with that at other sites in the body [45]. Studies in both human lung fibroblasts and human hepatic stellate cells have shown the ability of TLR-9 stimulation via CpG ODN to induce myofibroblast differentiation [28,46]. Furthermore, expression of TLR-9 on fibroblasts obtained from initial surgical lung biopsies of patients with IPF can distinguish patients with rapid disease progression (TLR-9-positive) from patients with a slower disease course (TLR-9-negative) [47]. Thus, it seems that in humans, TLR-9 stimulation may promote fibrotic reactions, whereas in mice, it may protect. Another important difference that may explain the improved outcomes in TLR-9-expressing mice is that mice have a larger repertoire of CpG-responsive hematopoietic cells. In mice, all DC subsets, B cells and macrophages respond to CpG ODN, whereas in humans, only plasmacytoid DCs and B cells respond [48]. Thus, it is likely that production of IFN-β in response to TLR-9 stimulation is greater in the murine system than it would be in humans. Collectively, these studies identify important differences between mice and men, and thus suggest that CpG therapy, although beneficial in rodent models of fibrosis, is unlikely to be beneficial for human treatment of IPF.

## Conclusions

TLR-9 signaling is protective during viral exacerbation of murine pulmonary fibrosis. This is probably due to increased levels of IFN-β, which limit fibroblast proliferation. There is no evidence that TLR-9 signaling during a viral exacerbation of pulmonary fibrosis alters alveolar epithelial-cell apoptosis. As TLR-9 signaling in human IPF fibroblasts appears to lead to a more profibrotic phenotype, these data highlight important differences between the human and mouse disease, and the limitations of our current animal models of pulmonary fibrosis.

## Methods

The Animal Use Committee at the University of Michigan (Ann Arbor, MI, USA) approved all protocols and experiments described.

### Mice

BALB/c and C57Bl/6 mice (Jackson Laboratories, Bar Harbor, ME, USA), aged 6 to 8 weeks old and matched for age and sex, were used. TLR-9^-/- ^mice backcrossed to Balb/c (University of Michigan, East Lansing, MI, USA) have been described previously [25].

### Fluoroscein isothiocyanate and bleomycin models of pulmonary fibrosis

Intratracheal (IT) inoculation of FITC (50 μl of a 2.8 mg/ml solution in saline) or bleomycin sulfate (0.035 per mouse in a 50 μl volume) (both from Sigma Chemical Co., St. Louis, MO, USA) was performed as described previously [49]. In C57Bl/6 mice, clinical grade bleomycin sulfate (Blenoxane; Bristol-Myers Squibb, New York, NY, USA) was utilized.

### Viral infection

Mice were anesthetized with ketamine and xylazine. Into 20 μl saline were suspended 5 ×10^4 ^PFU of γHV68 (American Type Culture Collection, Manassas, VA, USA), which were then delivered intranasally to each mouse. Sham infections consisted of intranasal delivery of 20 μl of saline.

### Lung collagen measurements

Total lung collagen measurements were made as described previously [16], using the Sircol collagen dye-binding assay (Accurate, Westbury, NY, USA). In some experiments, collagen content was estimated by hydroxyproline assay, as described previously [50].

### Histology

Lungs were inflated with 10% neutral-buffered formalin, and embedded in paraffin wax. Thin sections (5 μm) of lung were then stained with either hematoxylin and eosin or Masson's trichrome. For viral immunohistochemistry, frozen sections were prepared from infected mice, and γHV68 infection was detected using a rabbit polyclonal anti-γHV68 sera [51] (kindly provided by Dr Skip Virgin, Washington University School of Medicine, St. Louis, MO, USA) and detected with a goat anti-rabbit secondary antibody conjugated to alkaline phosphatase (Vectastain-ABC-AP kit for rabbit IgG (AK5001), Vector Laboratories, Burlingame, CA, USA).

TLR-9 immunohistochemistry was performed on wax-embedded tissues. The tissues were dewaxed and then blocked with H_2_O_2 _for antigen retrieval. They were incubated overnight at 4°C with a 1:50 of the primary antibody, a rabbit polyclonal antibody to TLR-9 (IMG-431; Imgenex Corp., San Diego, CA, USA). They were then incubated with biotin-labelled anti-rabbit IgG 1:500 (V1011; Vector Laboratories Inc., Burlingame, CA, USA) for 30 minutes at room temperature, followed by diaminobenzidine (catalog number 54-10-00; KPL Inc., Gaithersburg, MD, USA) for 10 minutes at room temperature. Control slides were stained with secondary antibody only.

TLR-9 immunofluorescence staining was performed on isolated cells using a mouse monoclonal primary antibody at 1:50 dilution (IMG-305A; Imgenex Corp) and Alexa Flour 555 goat anti-mouse IgG (catalog number A21424; Invitrogen Corp., Carlsbad, CA, USA). The slides were then mounted using anti-fade reagent (Prolong Gold; Invitrogen) with 4',6-diamidino-2-phenylindole (catalog number P36931, Invitrogen). Control slides were stained with secondary antibody only.

### ELISA

Cytokine levels were measured in whole-lung homogenates using commercial kits (R&D Systems; Minneapolis, MN, USA) according to the manufacturer's instructions. IFN-β was also measured using a commercial kit (PBL Biomedical Laboratories, Piscataway, NJ, USA).

### Flow cytometry

For fibrocyte enumeration, cells obtained by collagenase digestion were incubated for 15 minutes on ice with Fc block (clone 24G2; BD PharMingen, San Diego, CA, USA) before surface staining with CD45-PerCPCy5.5 (BD PharMingen) followed by fixation and permeabilization using a kit (Cytofix/cytoperm Kit; BD PharMingen) according to the manufacturer's instructions. Cells were then blocked with goat IgG before staining with collagen 1 (rabbit anti-mouse) (Rockland, Gilbertsville, PA, USA) followed by a goat anti-rabbit phycoerythrin-conjugated secondary antibody (Jackson Immunoresearch, West Grove, PA, USA). Rabbit IgG (Jackson Immunoresearch) was used as an isotype control in place of the anti-collagen antibody. Cells were analyzed on a flow cytometer (FACSCalibur; BD Biosciences, Mountain View, CA, USA).

For other leukocyte subsets, cells were stained with Fc block followed by surface staining for CD45, CD4, CD8, CD19, Gr-1 or DX-5 using directly conjugated antibodies (BD PharMingen).

### Isolation of alveolar epithelial cells

Type II alveolar epithelial cells were isolated and purified using a protocol published previously [52].

### Isolation of fibroblasts and fibrocytes

Mice were perfused with saline, and lung lobes were removed and minced with scissors. The minced tissue was cultured for 14 days in complete media to obtain a population of plastic-adherent mesenchymal cells. Fibroblasts were then negatively selected using anti-CD45-conjugated magnetic beads. Fibrocytes were the positively selected CD45 fraction.

### Isolation of dendritic cells

Bone marrow was cultured in complete media containing granulocyte-macrophage colony-stimulating factor (10 ng/ml) for 5 days. These cells were used as a positive control for TLR-9 expression by real-time RT-PCR.

### real-time reverse transcriptase PCR

Real-time RT-PCR was performed on a thermocycler (ABI Prism 7000; Applied Biosystems, Foster City, CA) using a protocol described previously [53). Genes of interest included the γHV68 lytic outer envelope glycoprotein B (gB), viral DNA polymerase, TLR-9 and IFN-β. Primer sequences and probes are listed in Table 1.

**Table 1:** Primer and Probe sequences for real-time RT-PCR

Gene	Oligo	Sequence 5'→3'
Glycoprotein B^a^	Forward	CGCTCATTACGGCCCAAA
	Reverse	ACCACGCCCTGGACAACTC
	Probe	TTGCCTATGACAAGCTGACCACCA
β-actin	Forward	CCGTGAAAAGATGACCCAGATC
	Reverse	CACAGCCTGGATGGCTACGT
	Probe	TTTGAGACCTTCAACACCCCCAGCCA
Interferon-β	Forward	TGACGGAGAAGATGCAGAAGAG
	Reverse	TGGAGTTCATCCAGGAGACGTA
	Probe	TGCCTTTGCCATCCAAGAGATGCTC
Toll-like receptor-9	Forward	GAGTACTTGATGTGGGTGGGAATT
	Reverse	GCCACATTCTATACAGGGATTGG
	Probe	CCGTCGCTGCGACCATGCC
DNA polymerase	Forward	ACAGCAGCTGGCCATAAAGG
	Reverse	TCCTGCCCTGGAAAGTGATG
	Probe	CCTCTGGAATGTTGCCTTGCCTCCA

^a^Gamma herpesvirus 68 open-reading frame 8.

### M30 staining

AECs were fixed and stained, according to manufacturer's instructions, with an antibody (M30 Cytodeath; Roche, Indianapolis, IN, USA) that detects a caspase cleavage product of cytokeratin 18 in epithelial cells.

### Caspase 3 western blotting

Western blots were performed as described previously using the follwoing antibodies: for cleaved caspase 3, a rabbit anti-human/mouse antibody (MAB835; R&D Systems) and for β-actin, a mouse monoclonal antibody (Sigma, cat# AC-74), with secondary antibodies for cleaved caspase 3 goat anti-rabbit (catalog number 31462; Pierce Protein Research Products, Thermo Fischer Scientific, Rockford, IL) and β-actin goat anti-mouse (catalog number 31432; Pierce Protein Research Products, Thermo Fischer Scientific, Rockford, IL).

### Proliferation assays

Fibroblasts were seeded at 5000 cells/well in 96-well flat-bottomed plates. ^3^H-thymidine (1 μCi) was added to each well during the final 16 hours. Cells were harvested onto glass-fiber filters, and the incorporated radioactivity was determined by scintillation counting. In some experiments, IFN-β (Cellsciences, Canton, MA, USA) was added during culture.

### *In vivo *CpG delivery

Specific-pathogen-free male C57BL/6 (wild-type (WT)) mice (Taconic, Germantown, NY, USA) 6 to 8 weeks of age were used. WT mice received 0.05 U of bleomycin sulfate (Blenoxane; Bristol-Meyers Pharmaceuticals, Evansville, IN, USA) dissolved in PBS (approximately 1.7 U/kg) via IT injection. Fourteen days later, all groups of mice were mildly anesthetized, and received a single bolus (50 μg) of CpG ODN (dissolved in sterile saline) by intranasal delivery. Groups of WT mice (n = 5 to 10 per timepoint) were monitored for survival. Mice were killed by cervical dislocation 28 days after the IT challenge with bleomycin, and whole-lung tissue was dissected for histological and biochemical analysis of hydroxyproline content. Untreated mice (n = 5) did not receive bleomycin, and this time point was designated as day 0.

### Statistical analyses

All calculations were performed using Prism 5.0 software (GraphPad Software, San Diego, CA, USA). Values are expressed as means *± *SEM. Two-sample *t*-tests were used for comparisons of the means when two groups were compared. One-way ANOVA was used for comparisons of three or more groups with Bonferroni or Dunnet post-hoc test analyses depending on whether all groups were compared with each other or all groups were compared with a single control group. *P *≤ 0.05 was considered significant.

## List of abbreviations

ELISA: enzyme-linked immunosorbent assay; PBS: phosphate-buffered saline.

## Competing interests

The authors declare that they have no competing interests.

## Authors' contributions

TRL contributed to the study concept and design, animal models, AEC isolation, real-time RT-PCR, ELISA, proliferation assays, data analysis, and drafting of the manuscript. SMC carried out real-time RT-PCR studies and ELISAs. GT carried out the CPG-ODN studies. JSS carried out the immunoblotting and cytokine ELISA assays. KMV assisted with cytokine ELISAs. UB participated in design of the study, DC isolation, apoptosis assays and intellectual content. CAW carried out the animal studies and fibroblast isolation. TAM performed flow cytometer analysis. GBT provided consultation in study concept and design, and manuscript preparation. CH participated in CpG-ODN studies and manuscript preparation. BBM participated in study concept and design, data analysis, and manuscript preparation. All authors have read and approved the final manuscript.

## Supplementary Material

Additional file 1 **Supplementary data and figures are included in a pdf file titled 'supplementary Figures'**. **Supplemental Figure 1:** TLR9 protects from gammaherpesvirus-induced exacerbation of FITC-induced fibrosis. A) Balb/c or TLR9-/- mice were injected with saline or 50 μl of a 2.8 mg/ml solution of FITC on day 0. On day 14, FITC-treated mice were mock-infected or infected with γHV-68. On day 21 lungs were harvested for collagen content determination by hydroxyproline assay (n = 3 per group in saline and n = 8 in other groups). B) H&E staining of lungs from FITC + γHV-68 groups on day 21. Representative of n = 3 other mice. **Supplemental Figure 2:** Immunohistochemistry demonstrating purity of AEC culture. AECs were isolated from mice treated with bleomycin + γHV-68 and were cultured on fibronectin-coated titer tek slides. Cells were then fixed and stained with e-cadherin or vimentin to demonstrate purity. Alkaline phosphatase detection shows that cells are >95% e-cadherin+ and less than 3% vimentin positive. **Supplemental Figure 3:** Balb/c and TLR9^-/- ^alveolar epithelial cells are equivalent in their ability to suppress fibroproliferation. AECs were isolated from Balb/or TLR9-/- naïve mice and were plated at 50,000 cells per well on fibronectin-coated 96 well plates. Fibroblasts at 5000 cells per well from Balb/c mice (panel A) or TLR9-/- mice (panel B) were added to each well and proliferation of fibroblasts was measured by ^3^H-thymidine incorporation after 24 h. AECs cultured alone had proliferation rates of less than 800 cpm., n = 12 per group. **Supplemental Figure 4:** TLR9^-/- ^mice have reduced T helper 2 responses during viral exacerbation of fibrosis. Lungs were harvested from Balb/c and TLR9-/- mice after bleomycin and γHV68 infection on days 0, 14, 17 and 21. Whole lung homogenates were made and the levels of IL-4, IL-13, INFγ, IL-12, IL-17 and TNFα were measured by ELISA. **Supplemental Figure 5:** No differences in TGFβ activation were seen between WT and TLR9-/- mice during a viral exacerbation of pulmonary fibrosis. Lungs were harvested from Balb/c and TLR9-/- mice treated in vivo with bleomcyin and γHV68 on day 21. Lung homogenates were acid-treated to activate total TGFβ and were then analyzed by ELISA, n = 5. Click here for file
