# Supplementary material for: TLR9-induced interferon β is associated with protection from gammaherpesvirus-induced exacerbation of lung fibrosis
Source: Fibrogenesis Tissue Repair. 2011 Aug 2;4:18. doi: 10.1186/1755-1536-4-18 (PMC3163187; doi:10.1186/1755-1536-4-18)
Supplement: Additional file 1 — Supplementary data and figures are included in a pdf file titled 'supplementary Figures'. Supplemental Figure 1: TLR9 protects from gammaherpesvirus-induced exacerbation of FITC-induced fibrosis. A) Balb/c or TLR9-/- mice were injected with saline or 50 μl of a 2.8 mg/ml solution of FITC on day 0. On day 14, FITC-treated mice were mock-infected or infected with γHV-68. On day 21 lungs were harvested for collagen content determination by hydroxyproline assay (n = 3 per group in saline and n = 8 in other groups). B) H&E staining of lungs from FITC + γHV-68 groups on day 21. Representative of n = 3 other mice. Supplemental Figure 2: Immunohistochemistry demonstrating purity of AEC culture. AECs were isolated from mice treated with bleomycin + γHV-68 and were cultured on fibronectin-coated titer tek slides. Cells were then fixed and stained with e-cadherin or vimentin to demonstrate purity. Alkaline phosphatase detection shows that cells are >95% e-cadherin+ and less than 3% vimentin positive. Supplemental Figure 3: Balb/c and TLR9-/- alveolar epithelial cells are equivalent in their ability to suppress fibroproliferation. AECs were isolated from Balb/or TLR9-/- naïve mice and were plated at 50,000 cells per well on fibronectin-coated 96 well plates. Fibroblasts at 5000 cells per well from Balb/c mice (panel A) or TLR9-/- mice (panel B) were added to each well and proliferation of fibroblasts was measured by 3H-thymidine incorporation after 24 h. AECs cultured alone had proliferation rates of less than 800 cpm., n = 12 per group. Supplemental Figure 4: TLR9-/- mice have reduced T helper 2 responses during viral exacerbation of fibrosis. Lungs were harvested from Balb/c and TLR9-/- mice after bleomycin and γHV68 infection on days 0, 14, 17 and 21. Whole lung homogenates were made and the levels of IL-4, IL-13, INFγ, IL-12, IL-17 and TNFα were measured by ELISA. Supplemental Figure 5: No differences in TGFβ activation were seen between WT and TLR9-/- mice during a viral e [file 1755-1536-4-18-S1.PDF]

A

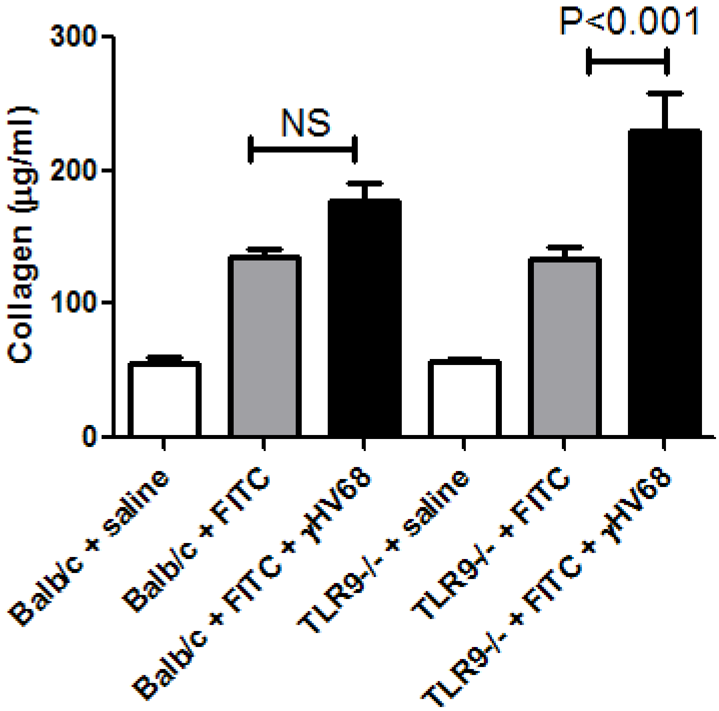

B

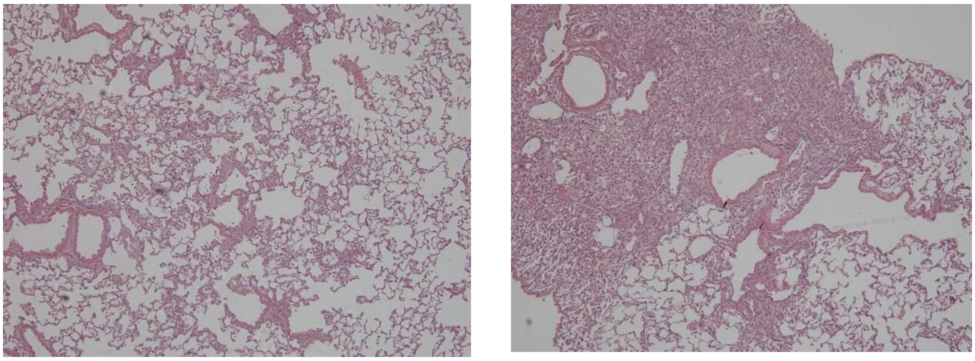

Balb/c + FITC + γHV-68 **TLR9-/- + FITC + γHV-68**

Supplemental Figure 1) A) Balb/c or TLR9-/- mice were injected with saline or 50µl of a 2.8 mg/ml solution of FITC on day 0. On day 14, FITC-treated mice were mock-infected or infected with γHV-68. On day 21 lungs were harvested for collagen content determination by hydroxyproline assay (n=3 per group in saline and n=8 in other groups). B) H&E staining of lungs from FITC + γHV-68 groups on day 21. Representative of n=3 other mice.

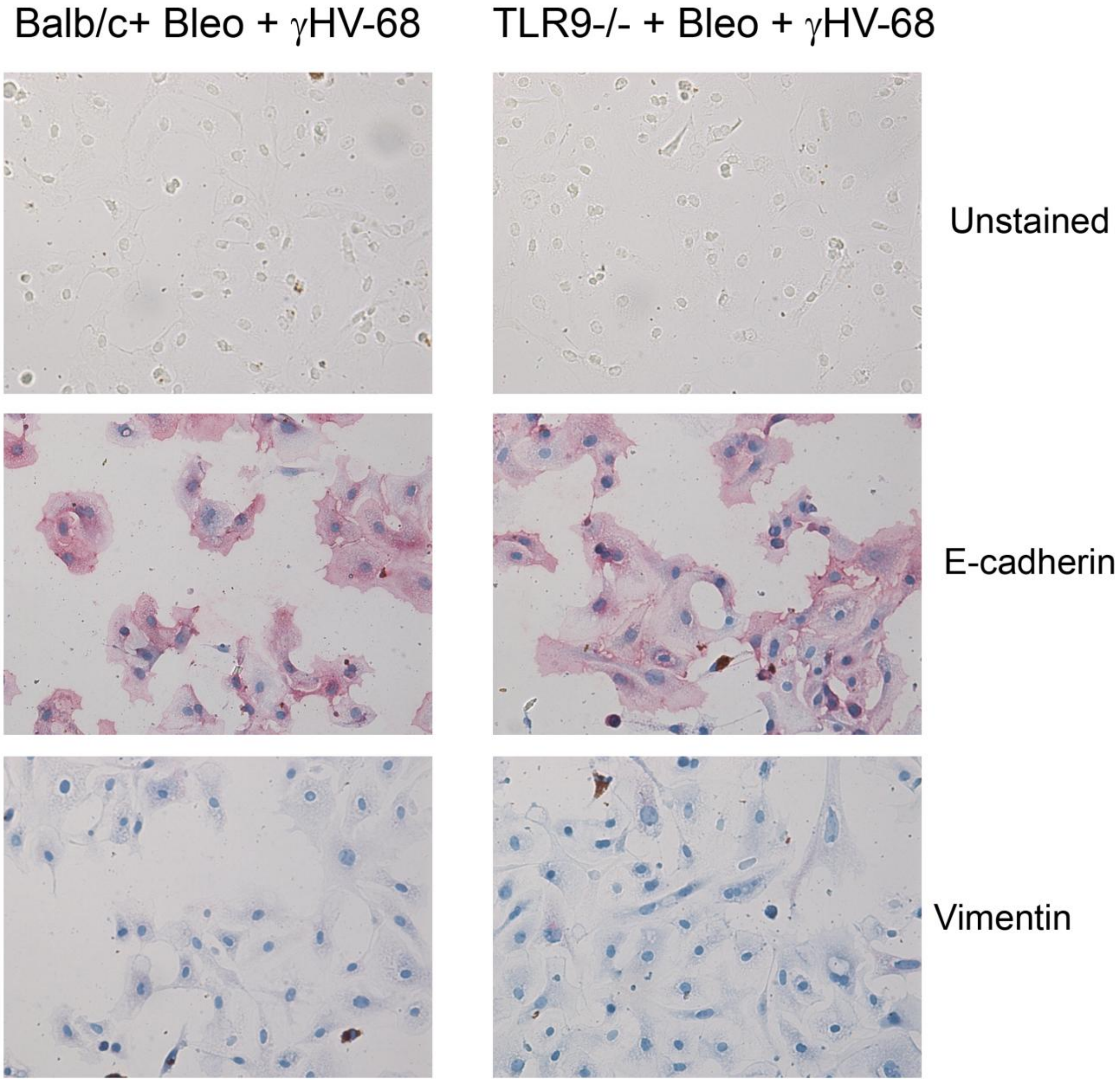

Supplemental Figure 2) AECs were isolated from mice treated with bleomycin + γHV-68 and were cultured on fibronectin-coated titer tek slides. Cells were then fixed and stained with e-cadherin or vimentin to demonstrate purity. Alkaline phosphatase detection shows that cells are >95% e-cadherin+ and less than 3% vimentin positive.

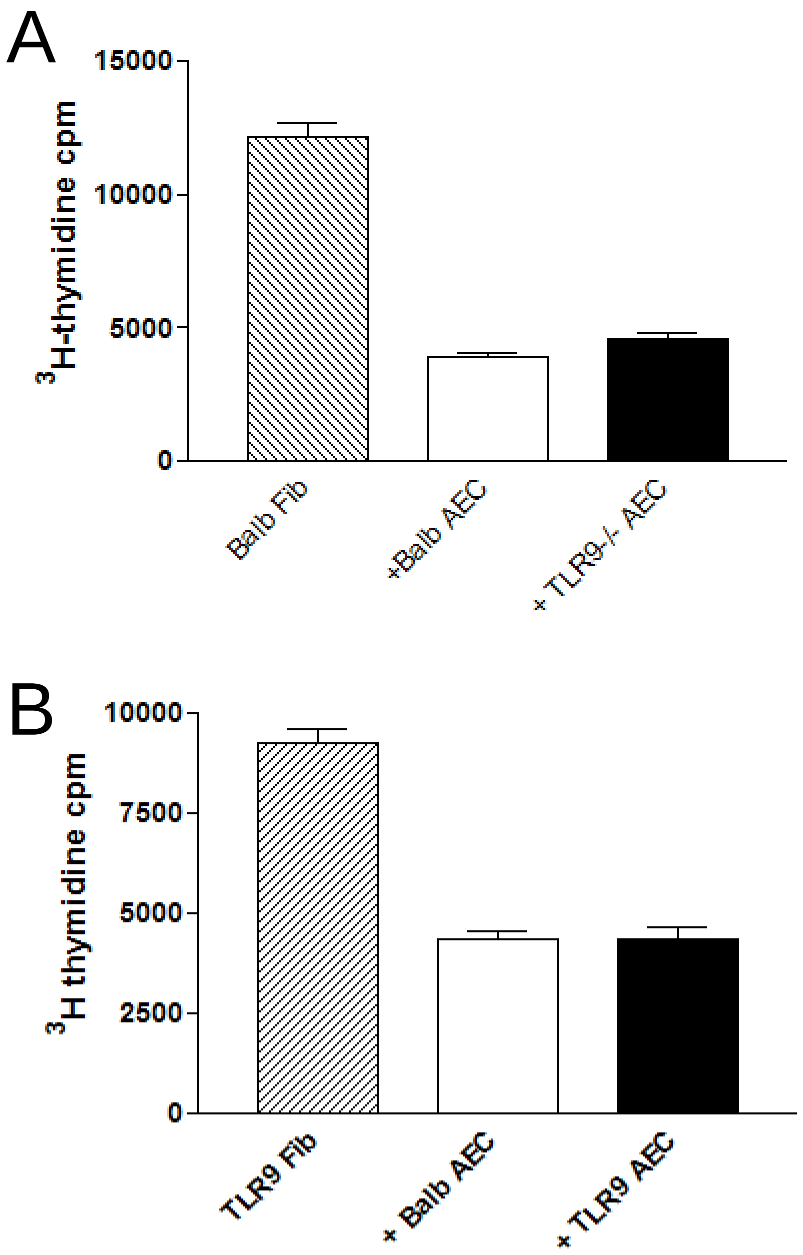

Supplemental Figure 3) AECs were isolated from Balb/or TLR9-/- naïve mice and were plated at 50,000 cells per well on fibronectin-coated 96 well plates. Fibroblasts at 5000 cells per well from Balb/c mice (panel A) or TLR9-/- mice (panel B) were added to each well and proliferation of fibroblasts was measured by ^{3}H-thymidine incorporation after 24 h. AECs cultured alone had proliferation rates of less than 800 cpm., n=12 per group.

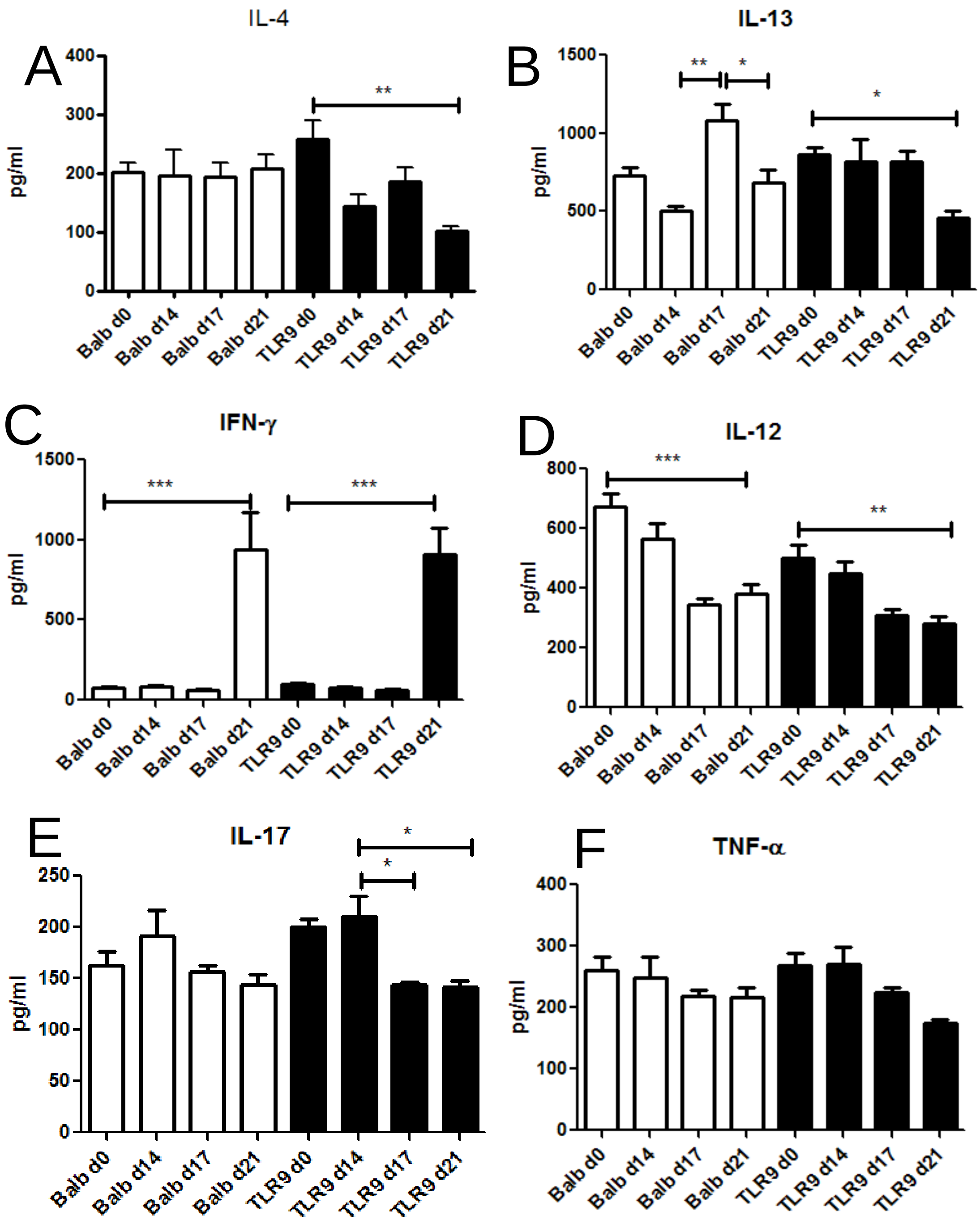

Supplemental Figure 4) Lungs were harvested from Balb/c and TLR9-/- mice after bleomycin and γHV68 infection on days 0, 14, 17 and 21. Whole lung homogenates were made and the levels of IL-4, IL-13, INFγ, IL-12, IL-17 and TNFα were measured by ELISA.

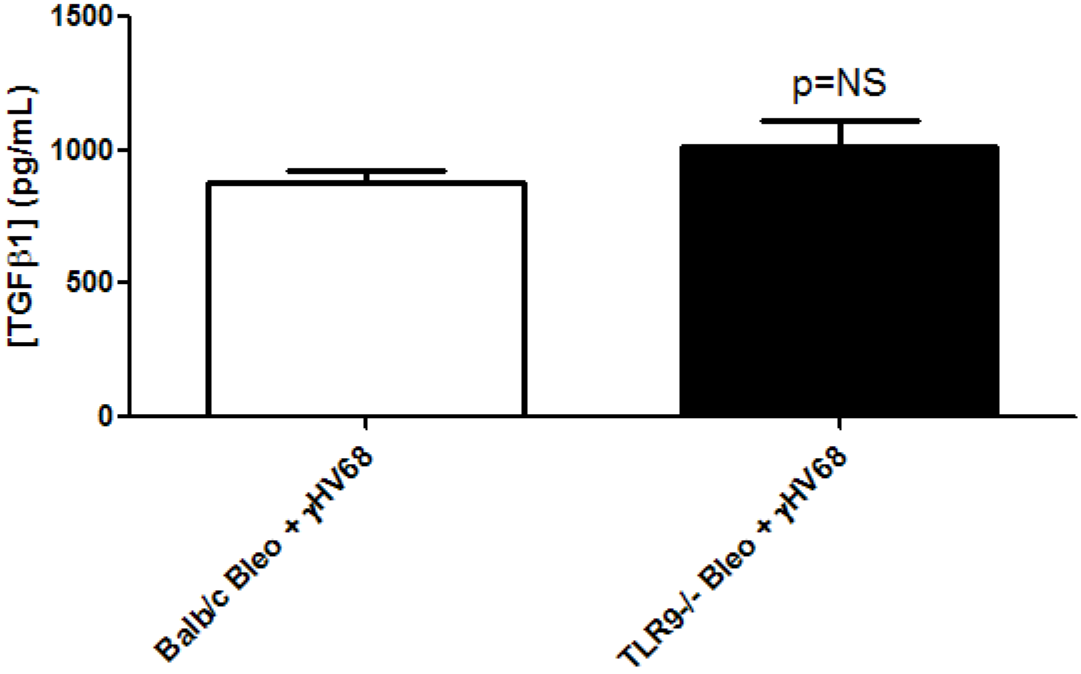

Supplemental Figure 5) Lungs were harvested from Balb/c and TLR9-/- mice treated in vivo with bleomcyin and γHV68 on day 21. Lung homogenates were acid-treated to activate total TGFβ and were then analyzed by ELISA, n=5.
